# Progress in Surgery

**Published:** 1897-02-13

**Authors:** 


### PROGRESS IN SURGERY.

### SURGERY OF THE ŒSOPHAGUS.

(*Continued from page* 200.)

**Non-Malignant Pyloric Stricture** has been treated in several cases by Bond[13] by means of Loreta's operation, pyloro-plasty, and gastro-jejunostomy. He considers it clear that ulceration of the mucous membrane, either on the gastric or duodenal side of the pylorus, is the chief cause of pyloric cicatricial stricture, and it is doubtful whether mere external adhesions to the liver or neighbouring organs, apart from any process which involves the mucous membrane, are sufficient to cause the deposition of cicatricial tissue in the annular form which is characteristic of these cases. If the ulceration has ceased and the pylorus is left as a tightly closed cicatricial ring, pyloric stretching by Loreta's method is unsatisfactory from liability to recurrence, and the choice lies between pyloroplasty and gastro-jejunostomy, and probably the latter is the better. When, however, ulceration is still progressive, even though the stenosis has not reached an extreme degree, it will be found that gastro-jejunostomy has several advantages over the plastic operation. The artificial opening is made through healthy tissues, and so is more likely to heal, and less likely to contract. Moreover, the pylorus is set at rest, and the ulcer placed in the most favourable conditions for healing. Operation should not be delayed until the patient is suffering from starvation, but should be done somewhat early in the course of the disease. Lejars[14] has had a successful case of gastro-enterostomy by means of Murphy's button for rapid pyloric stenosis following on the ingestion of half a glassful of dilute hydrochloric acid. The case is interesting because of the limitation of the burns to the stomach, the œsophagus not being affected by the passage through it of the caustic fluid; and because of the great rapidity of development of pyloric stenosis and its resulting symptoms. Somewhat allied to this condition of pyloric stenosis is an interesting case of Watson,[15] in which an hour-glass contraction of the stomach occurred from cicatrisation of a gastric ulcer. Resection of the cicatrix seemed out of the question, and it seemed to the author that it would be better to establish an anastomosis between the upper and lower parts of the stomach, instead of between the stomach and intestine, and in this way to retain the useful function of the pylorus. This was accordingly done, and the patient made an excellent recovery.

**Gastric Ulcer** heals spontaneously, according to Welch, in not less than 85 per cent. of the whole. The indications for operation according to Parker[16] are: (1) Stenosis of the pylorus, for which pylorectomy, pyloroplasty, or excision of the ulcer and gastro-enterostomy are indicated. (2) Repeated and uncontrollable hæmorrhages. Mikulicz, Küster, and Roux have saved patients by laparotomy, together with suture, excision, or cauterisation of the base of the ulcer.

(3) Perigastritis, localised peritonitis, and the results from the cicatricial adhesions of the stomach to neighbouring organs, and the interference with its functions. (4) Gastrectasia. Eighty per cent. of ulcers followed by perforation are situated on the anterior aspect of the stomach—a situation which increases the danger of a general peritonitis, although the chances of rational treatment are more likely to be satisfactory. An ulcer in this situation may be followed by three phases: (1) Most frequent, a general peritonitis. (2) An opening into a neighbouring viscus, and the formation of a fistula. (3) An adhesive peritonitis and encapsulation either between the liver and the diaphragm, the liver and the stomach, or the spleen and the stomach. Two symptoms may be considered diagnostic: (1) A sharp, "tearing" pain in the epigastrum; sometimes, but not always, preceded by signs of ulcer of the stomach. (2) Signs of general peritonitis preceded by signs of perforation, or by those of a localised peritonitis, with encapsulation at least for a certain period. Weir and Foote[17] quote Barling as saying that 95 per cent. of patients having perforations, unless operated upon, die. Operations performed under twelve hours after perforation show a mortality of 39 per cent.; in the next twelve hours, of 76 per cent.; and after twenty-four hours, of 87 per cent. The authors recommend, where practicable, simple suture by Lembert's method. Cumston.[18] however, prefers excision of the ulcer before suture. Thomson[19] supports the conclusions of Weir mentioned above. He lays stress on the difficulty of diagnosis of perforation, and reports a case in which the symptoms so closely resembled perforation that an operation was performed, but the peritoneal cavity was quite clear. This nervous simulation of perforation generally occurs in young adult women. An internal hæmorrhage, in the absence of vomiting, may also resemble symptoms of perforation, and the author thinks it would be good practice to pass a tube into the stomach and acquire accurate information as to its contents, for in perforation blood is not present in the stomach. Kukula[20] says that in addition to Küster's (pyloric stenosis, perforative peritonitis, and uncontrollable hæmorrhage) indications for operation, we should recognise another, viz., a painful swelling, or an apparent tumour—the result of adhesions. Other cases submitted to operation, successfully and unsuccessfully, are reported by Dent,[21] Jowers,[22] Barker,[23] Hofmeister,[24] Morgan,[25] Curtis,[26] Von Noorden,[27] Bennett,[28] and others.

13 Lancet, July 25th, 1896. 14 Med. Week, June 19th, 1896. 15 Therap. Gazette, Aug. 15th, 1896, and Bost. Med. and Surg. Journ., Ap. 2nd, 1896. 16 Annals of Surgery, June, 1896. 17 Med. Chronicle, June, 1896, and Med. News, April 25th and May 2nd, 1896. 18 Edin. Med. Journ., July, 1896, and Boston Med. and Surg. Journ., Ap. 23rd, 1896. 19 Lancet, July 4th, 1896. 20 Amer. Med.-Surg. Bulletin, Sept. 26th, 1896, and Wein Klin. Rundsch., No. 21, 1896. 21 Lancet, June 20th, 1896. 22 Ibidem. 23 Clinic Journ., April 8th, 1896. 24 Med. Week, May 29th, 1896. 25 Brit. Med. Journ., June 13th, 1896. 26 Annals of Surg., Aug., 1896. 27 Brit. Med. Journ., Oct. 3rd, 1896, and Münch. Med. Woch, Sept. 1st, 1896. 28 Lancet, Aug. 1st, 1896.

